# Geographic Disparities in Stroke Action Awareness in China: A National Survey

**DOI:** 10.1002/cns.71056

**Published:** 2026-08-04

**Authors:** Jing Yuan, Renyu Liu, Yu Feng, Yao Wang, Shujian Zhang, Qing Li, Xunming Ji, Jing Zhao

**Affiliations:** ^1^ Institute of Chinese Medical Sciences, Centre for Pharmaceutical Regulatory Sciences & Faculty of Health Sciences University of Macau Macao SAR China; ^2^ Department of Anesthesiology and Critical Care Perelman School of Medicine at the University of Pennsylvania Philadelphia Pennsylvania USA; ^3^ Department of Neurology Affiliated Hospital of Xuzhou Medical University Xuzhou Jingsu Province People's Republic of China; ^4^ Department of Neurology Sixian People's Hospital Suzhou Anhui Province People's Republic of China; ^5^ Department of Neurology Fangcheng County People's Hospital Nanyang Henan Province People's Republic of China; ^6^ Department of Neurology People's Hospital of Xinjiang Uygur Autonomous Region Urumqi People's Republic of China; ^7^ Xinjiang Clinical Research Center for Stroke and Neurological Rare Disease Urumqi People's Republic of China; ^8^ Department of Neurosurgery Xuanwu Hospital, Capital Medical University Beijing People's Republic of China; ^9^ Beijing Key Laboratory of Hypoxic Conditioning Translational Medicine Xuanwu Hospital, Capital Medical University People's Republic of China; ^10^ Department of Neurology Minhang Hospital, Fudan University Shanghai China

**Keywords:** prehospital delay, stroke action awareness, stroke belt, survey

## Abstract

**Objective:**

Stroke incidence and mortality are higher among adults in northern and western China, commonly referred to as the “Stroke Belt.” However, few studies have compared stroke action awareness between Stroke Belt and non–Stroke Belt regions at the national level. This study aimed to evaluate stroke action awareness—including recognition of symptoms and appropriate emergency responses—among community residents in China, with comparisons between Stroke Belt and non‐Stroke Belt regions.

**Methods:**

We conducted a cross‐sectional, questionnaire‐based survey of community residents across China to assess knowledge of stroke symptoms and appropriate responses to suspected stroke events.

**Results:**

A total of 6181 residents were included in the final analysis, with 2037 living in Stroke Belt regions and 4144 in non‐Stroke Belt regions. Overall, 19.07% (*n* = 1179) were unaware of any stroke symptoms, 17.96% (*n* = 1110) recognized one symptom, 11.97% (*n* = 740) recognized two symptoms, and 50.99% (*n* = 3152) recognized all three. Additionally, 77.59% (*n* = 4796) identified calling emergency medical services (EMS) as the correct response. Multivariable logistic regression showed that residents of the Stroke Belt had significantly lower odds of recognizing all three symptoms (adjusted odds ratio [aOR]: 0.50; 95% CI: 0.44–0.56; *p* < 0.001), calling EMS (aOR: 0.62; 95% CI: 0.54–0.70; *p* < 0.001), and both recognizing all three symptoms and calling EMS (aOR: 0.51; 95% CI: 0.45–0.58; *p* < 0.001).

**Conclusion:**

Stroke action awareness remains notably low among community residents in China, with the lowest levels observed in the Stroke Belt. Targeted public health interventions are urgently needed to improve symptom recognition and promote timely action for emergency stroke care, especially in high‐risk regions such as the Stroke Belt.

## Graphical abstract

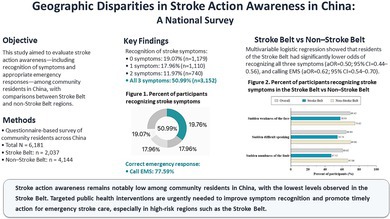

## 1 Introduction

Stroke remains the leading cause of mortality and disability worldwide [1]. Although mortality rates for patients with acute ischemic stroke (AIS) have shown a downward trend, these patients continue to experience significant levels of disability and loss of disability‐adjusted life years (DALYs) [1, 2]. Reperfusion therapies, such as tissue plasminogen activator (tPA) and endovascular therapy (EVT), have demonstrated significant benefits for improving functional outcomes, but they are highly time‐sensitive [3, 4, 5]. Stroke patients are expected to arrive at the hospital within 4.5 h to be eligible for the tPA [6]. Nevertheless, delayed presentation to the hospital represents a significant barrier to the widespread application of tPA therapy in stroke care [3, 4], particularly in China, where a small percentage of AIS patients receive tPA [7], contributing to persistently high stroke‐related disability and mortality in China [3].

Knowledge of stroke symptoms is only the first step; stroke action awareness, which involves recognizing stroke signs and taking appropriate action [8], is critical for determining whether patients can benefit from life‐saving reperfusion therapies. A survey conducted in 2011 revealed that only 1 in 5 community residents in China could identify stroke symptoms and act appropriately [9]. To enhance stroke action awareness, the Stroke 1‐2‐0 tool was specifically developed for the Chinese population by adapting the FAST (Face, Arm, Speech, Time) acronym, which has been the standard in English‐speaking countries [10, 11, 12]. Since 2017, after the educational campaigns have been implemented to promote Stroke 1‐2‐0 [13], there is limited data examining the prevalence and characteristics of community residents who lack awareness of these symptoms and emergency responses at the national level [9, 14, 15]. For instance, the FAST‐RIGHT study surveyed the stroke symptom awareness in early 2017 [14, 15]. Recent studies focused primarily on symptom recognition within specific urban or provincial populations [16]. Hence, the current status of stroke action awareness remains unclear at the national level. Furthermore, the relationship between sociodemographic and geographic factors and stroke action awareness remains largely unexplored in China, making such information crucial for identifying subgroups that require targeted educational efforts.

Recent studies have indicated a higher incidence of stroke and mortality rates among adults residing in northern and western China [17], regions commonly referred to as the “Stroke Belt” [18, 19], which is comparable to the U.S. “Stroke Belt” in terms of geographic, socioeconomic, and health‐system disparities. However, there are limited studies comparing stroke awareness among adults living in the Stroke Belt versus those in non‐Stroke Belt regions. Therefore, this study aimed to evaluate stroke action awareness—including recognition of symptoms and appropriate emergency responses—among community residents in China, comparing Stroke Belt and non‐Stroke Belt regions while accounting for various sociodemographic and geographic factors.

## 2 Methods

### 2.1 Study Design and Participants

We employed a cross‐sectional study design. Community residents were invited to participate in a questionnaire‐based survey assessing knowledge of stroke symptoms and appropriate responses to suspected strokes in China. The anonymous online survey was administered across China from October 1, 2021 to January 31, 2023. Participation was voluntary and anonymous, and no incentives were provided. The reporting of this study adhered to the Checklist for Reporting Results of Internet E‐Surveys (CHERRIES).

### 2.2 Ethical Statement

This study was approved by the Institutional Review Boards of Minhang Hospital of Fudan University (Approval No. 2020‐039‐01K). Informed consent was waived as this study involved only de‐identified data and posed minimal risk to participants. All procedures complied with applicable ethical standards and the Declaration of Helsinki.

### 2.3 Survey Questionnaire Development

The questionnaire was developed in Chinese to ensure clarity for the general population. The items were derived from our previous research, literature reviews, and input from clinical experts. The clarity and content validity of the questionnaire have been reviewed and approved by the clinical experts. We also piloted the questionnaire among the elderly to ensure the survey questions were easy to understand. The questionnaire consists of four parts. The first part collected sociodemographic and geographic information, including age, sex, education, occupation, residence, and stroke risk factors. The second part assessed individuals' awareness of stroke symptoms and their responses to perceived strokes. The third part evaluated participants' knowledge of acute stroke therapies, while the fourth part investigated individuals' perceived needs for health education to promote stroke awareness.

### 2.4 Participant Recruitment and Questionnaire Administration

The questionnaire was distributed through convenience and snowball sampling [20, 21, 22]. Convenience sampling was used to initiate recruitment across the predefined Stroke Belt and non‐Stroke Belt regions, and snowball sampling was then used to broaden community reach. Stroke Belt regions include 1 municipality, 4 provinces, and 4 autonomous regions. We first reached out to a large number of healthcare providers and community workers willing to distribute the online questionnaire via QR codes or hyperlinks. Then they were trained in distributing the questionnaire, sent invitations to existing contact networks, promoted it on social networks, and were asked to share the questionnaire with other locals in their social networks. This approach was intended to improve geographic coverage rather than to generate a probability‐based sample. All respondents were also asked to share the questionnaire.

To prevent multiple submissions from respondents, Wenjuanxing (wjx.cn) enforces one submission per account/device and uses IP and device‐fingerprint checks to flag duplicates. We also excluded abnormal or duplicate entries. A total of 8000 residents were invited to participate, of whom 6240 responded, resulting in a response rate of 78%. The questionnaire was considered valid if it met the following criteria: (1) all questions were answered, (2) the respondent was over 18 years old, and (3) the questionnaire was completed within 5 min to ensure that the participants did not search online or ask for help.

### 2.5 Study Variables

Awareness of stroke symptoms was evaluated based on participants' responses to the question, “Which of the following do you identify as signs of a stroke?” We assessed recognition of the three most common symptoms that were specifically included in the Stroke 1–2‐0 tool: (1) sudden numbness or weakness of the limb; (2) sudden weakness of the face; and (3) sudden difficulty speaking [10]. Participants' responses were analyzed individually and subsequently categorized into four mutually exclusive groups based on the number of symptoms they recognized: none, one sign, two signs, and three signs.

To assess awareness of the need to call emergency medical services (EMS) in response to a perceived stroke, participants were asked, “What is the best thing to do when someone is having a stroke?” The response options included calling the emergency number (1–2‐0) or an ambulance, driving to the hospital, consulting with a physician, seeking assistance from a spouse or family member, and other alternatives [23]. The responses were dichotomized into two groups: calling the emergency number and all other options. Additionally, we evaluated awareness of the stroke center map by asking, “Do you know the map for stroke centers?” The response options included very familiar, heard of, and unknown.

Awareness of acute stroke therapy was assessed through individuals' responses to the question, “What is the most effective therapy for treating stroke?” The responses included tPA and EVT [24]. Additionally, we included the following questions to further evaluate participants' awareness of the urgency of accessing these life‐saving therapies: “Do you know that you should seek care within 4.5 hours of stroke onset to receive tPA?” and “Do you know that you should seek care within 6.0 hours of onset to receive EVT?” The response options consisted of “very familiar,” “heard of,” and “unknown”, and were analyzed individually.

To better understand community residents' needs for future public health promotion, participants were asked the question, “What is your preferred source for acquiring knowledge about strokes?” The responses included healthcare providers, magazines, booklets, newspapers, radio broadcasts, short videos, the Internet, social media, and television.

The stroke belt was defined as participants' self‐reported residency in 1 municipality, 4 provinces, and 4 autonomous regions, including Heilongjiang, Tibet, Jilin, Liaoning, Xinjiang, Hebei, Inner Mongolia, Beijing, and Ningxia, as identified in previous studies [17]. Non‐Stroke Belt regions included 3 municipalities, 16 provinces, and 1 autonomous region. The schematic map of the stroke belt and the non‐stroke belt was shown in Figure S1. Additional study variables included in this analysis were age (i.e., 18–44 years, 45–59 years, 60–74 years, or 75 years and above), sex (i.e., male or female), family income (i.e., < 10,000 CNY, 10,000–50,000 CNY, > 50,000 CNY), education level (i.e., elementary school, high school, college, or graduate school), occupation (i.e., employees of state‐owned firms/institutions, employees of private firms/institutions, self‐employed, professional technical personnel, farmers or herders, unemployed, or others), prior history of stroke or transient ischemic attack (TIA), family history of stroke, and other stroke risk factors (e.g., hypertension and diabetes). The occupation was classified based on the Ministry of Human Resources and Social Security of China. Participants were classified as having a higher risk of stroke if they presented with one or more risk factors, following the American Stroke Association's stroke risk assessment tool.

### 2.6 Statistical Analysis

The data were analyzed using SAS 9.4. Descriptive analysis was conducted for each study variable. Continuous variables (i.e., age) are presented as mean ± standard deviation (SD) when normally distributed, and comparisons were made using Student's *t*‐test. Categorical variables are expressed as counts and percentages, and comparisons were performed using the chi‐squared test. To examine the associations between awareness of stroke symptoms and various sociodemographic and geographic factors, a logistic regression model was employed to estimate odds ratios (ORs) with 95% confidence intervals (CIs). Multivariable models adjusted for age group, sex, education, annual household income, Stroke Belt residence, prior stroke/TIA, family history of stroke, and presence of stroke risk factors (hypertension, diabetes). A *p*‐value of less than 0.05 was deemed to indicate statistical significance.

## 3 Results

### 3.1 Characteristics of Survey Participants

A total of 6240 residents completed the questionnaire, and 59 were excluded from the analysis because of missing data or being under 18 years old. Hence, a total of 6181 residents were included in the final analysis; 2037 lived in the Stroke Belt regions, and 4144 lived in the non‐Stroke Belt regions.

The majority of the participants were female (Table 1; *n* = 3597; 58.19%), with an average age of 48.15 ± 15.73 years. A total of 1459 participants (23.6%) resided in Northern China, 582 (9.42%) in Northeast China, 555 (8.98%) in Northwest China, 359 (5.81%) in Southwest China, 1592 (25.76%) in Eastern China, 467 (7.56%) in Southern China, and 1167 (18.88%) in Central China. Most of the participants had high school (*n* = 2351; 38.04%) or college/graduate education (*n* = 1896; 30.67%). The majority of individuals had an annual income of < 10,000 CNY (*n* = 1394; 22.55%) or 10,000–50,000 CNY (*n* = 2380; 38.51%). Participants residing in the Stroke Belt regions were more likely to be at an older age, have a lower education level, and have higher risk factors for stroke.

**TABLE 1:** Baseline characteristics of survey participants.

Characteristic	Overall (*n* = 6181)	Stroke belt (*n* = 2037)^a^	Non‐stroke belt (*n* = 4144)	*p*
Age, year				< 0.001
18–44	2646 (42.81%)	721 (35.40%)	1925 (46.45%)	
45–59	1921 (31.08%)	644 (31.62%)	1277 (30.82%)	
60–74	1344 (21.74%)	575 (28.23%)	769 (18.56%)	
75+	270 (4.37%)	97 (4.76%)	173 (4.17%)	
Sex				0.339
Men	2584 (41.81%)	869 (42.66%)	1715 (41.39%)	
Women	3597 (58.19%)	1168 (57.34%)	2429 (58.61%)	
Education				< 0.001
Elementary school	276 (4.47%)	97 (4.76%)	179 (4.32%)	
Middle school	1658 (26.82%)	652 (32.01%)	1006 (24.28%)	
High or vocational school	2351 (38.04%)	801 (39.32%)	1550 (37.4%)	
College or above	1896 (30.67%)	487 (23.91%)	1409 (34%)	
Marital status				< 0.001
Married	5068 (81.99%)	1668 (81.89%)	3400 (82.05%)	
Divorced/widowed	419 (6.78%)	197 (9.67%)	222 (5.36%)	
Never married	694 (11.23%)	172 (8.44%)	522 (12.60%)	
Occupation				< 0.001
Employees of state‐owned firms/institutions	1505 (24.35%)	530 (26.02%)	975 (23.53%)	
Employees of Private firms	709 (11.47%)	311 (15.27%)	398 (9.60%)	
Self‐employed	597 (9.66%)	243 (11.93%)	354 (8.54%)	
Professional technical personnel	1322 (21.39%)	241 (11.83%)	1081 (26.09%)	
Farmers or herders	810 (13.10%)	310 (15.22%)	500 (12.07%)	
Unemployed	344 (5.57%)	133 (6.53%)	211 (5.09%)	
Others	894 (14.46%)	269 (13.21%)	625 (15.08%)	
Annual income, CNY				0.222
< 10,000	1394 (22.55%)	437 (21.45%)	957 (23.09%)	
10,000‐50,000	2380 (38.51%)	811 (39.81%)	1569 (37.86%)	
> 50,000	2407 (38.94%)	789 (38.73%)	1618 (39.04%)	
Any stroke risk factors	3826 (61.90%)			
Family history of stroke	913 (14.77%)	373 (18.31%)	540 (13.03%)	< 0.001
Prior stroke or TIA	580 (9.38%)	258 (12.67%)	322 (7.77%)	< 0.001
Hypertension	1658 (26.82%)	628 (30.83%)	1030 (24.86%)	< 0.001
Diabetes	760 (12.30%)	313 (15.37%)	447 (10.79%)	< 0.001
Heart	550 (8.90%)	273 (13.40%)	277 (6.68%)	< 0.001
Hyperlipidemia	593 (9.59%)	218 (10.70%)	375 (9.05%)	0.038
Obesity	728 (11.78%)	236 (11.59%)	492 (11.87%)	0.742
Cigarette smoking	823 (13.31%)	303 (14.87%)	520 (12.55%)	0.011
Lack of exercise	1372 (22.20%)	377 (18.51%)	995 (24.01%)	< 0.001

Abbreviations: CNY, Chinese Yuan; TIA, transient ischemic attack.

^a^ The Stroke Belt regions Heilongjiang, Tibet, Jilin, Liaoning, Xinjiang, Hebei, Inner Mongolia, Beijing, and Ningxia.

### 3.2 Symptom Awareness

**FIGURE 1:**
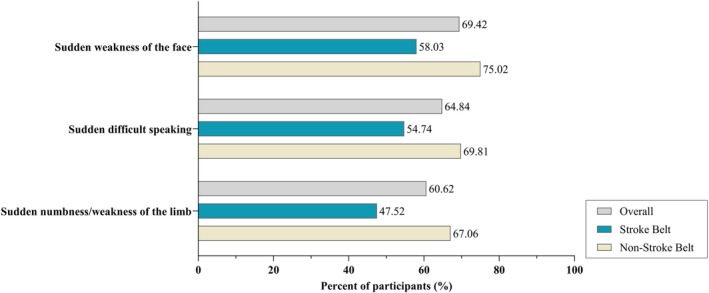
Proportion (%) of individuals aware of stroke symptoms.

Among the 6181 community residents who participated in this survey, 19.07% (*n* = 1179) were not aware of any stroke signs, 17.96% (*n* = 1110) recognized only 1 sign, 11.97% (*n* = 740) recognized 2 signs, and 50.99% (*n* = 3152) recognized all 3 signs (Table 2). The proportion of participants who recognized all three signs was lower in the Stroke Belt regions than in the non‐Stroke Belt regions (36.87% vs. 57.94%; *p* < 0.001). In contrast, the percentage of participants unaware of any stroke signs was higher in the Stroke Belt regions than in the non‐Stroke Belt regions (26.76% vs. 15.30%; *p* < 0.001). As shown in Figure 1, most participants considered the sudden weakness of the face a stroke symptom (*n* = 4291; 69.42%), followed by difficulty speaking (*n* = 4008; 64.84%), and sudden numbness or weakness of the limb (*n* = 3747; 60.62%). With respect to each stroke symptom, the proportion of participants who answered correctly was lower in the Stroke Belt than in the non‐Stroke Belt regions, including sudden numbness/weakness of the limb (47.52% vs. 67.06%; *p* < 0.001), sudden difficulty speaking (54.74% vs. 69.81%; *p* < 0.001), and sudden weakness of the face (58.03% vs. 75.02%; *p* < 0.001).

**TABLE 2:** Percentages of community residents who knew stroke signs and appropriate action to take in the event of a stroke.

Characteristic	Overall						Stroke belt^a^						Non‐stroke belt					
	Know no signs	Know one sign	Know two signs	Know all three signs	Knows to call EMS	Knows all three signs and to call EMS	Know no signs	Know one sign	Know two signs	Know all three signs	Knows to call EMS	Knows all three signs and to call EMS	Know no signs	Know one sign	Know two signs	Know all three signs	Knows to call EMS	Knows all three signs and to call EMS
Overall	19.07	17.96	11.97	50.99	77.59	43.83	26.76	23.07	13.30	36.87	70.30	30.19	15.30	15.44	11.32	57.94	81.18	50.53
Age, year																		
18–44	14.97	14.70	9.33	61.00	82.28	53.55	21.91	17.61	12.48	47.99	75.31	40.08	12.36	13.61	8.16	65.87	84.88	58.60
45–59	19.10	17.54	12.60	50.75	78.81	43.57	28.88	22.83	11.49	36.80	73.91	30.75	14.17	14.88	13.16	57.79	81.28	50.04
60–74	25.67	23.29	15.92	35.12	69.49	28.20	31.13	28.35	15.13	25.39	64.87	19.13	21.59	19.51	16.51	42.39	72.95	34.98
75+	26.30	26.30	13.70	33.70	63.33	28.15	22.68	34.02	20.62	22.68	41.24	18.56	28.32	21.97	9.83	39.88	75.72	33.53
Sex																		
Men	21.48	19.20	13.51	45.82	74.50	38.89	28.08	23.71	14.50	33.72	65.48	27.27	18.13	16.91	13.00	51.95	79.07	44.78
Women	17.35	17.07	10.87	54.71	79.82	47.37	25.77	22.60	12.41	39.21	73.89	32.36	13.30	14.41	10.13	62.17	82.67	54.59
Education																		
Elementary school	37.68	23.19	11.23	27.90	56.52	20.65	40.21	28.87	8.25	22.68	46.39	14.43	36.31	20.11	12.85	30.73	62.01	24.02
Middle school	26.96	21.83	17.13	34.08	72.68	28.47	32.67	26.07	14.26	26.99	67.02	21.32	23.26	19.09	18.99	38.67	76.34	33.10
High school	17.78	19.65	12.38	50.19	78.48	42.79	24.97	25.22	13.73	36.08	73.16	30.09	14.06	16.77	11.68	57.48	81.23	49.35
College or above	11.08	11.71	7.07	70.15	83.86	61.92	19.10	14.37	12.32	54.21	74.74	45.38	8.30	10.79	5.25	75.66	87.01	67.64
Marriage																		
Married	18.86	17.58	12.13	51.42	79.30	44.63	27.22	22.78	13.13	36.87	73.38	30.82	14.76	15.03	11.65	58.56	82.21	51.41
Divorced/widowed	24.82	22.91	16.47	35.80	58.23	26.73	23.86	27.92	17.77	30.46	46.19	20.81	25.68	18.47	15.32	40.54	68.92	31.98
Never married	17.15	17.72	8.07	57.06	76.80	48.27	25.58	20.35	9.88	44.19	68.02	34.88	14.37	16.86	7.47	61.30	79.69	52.68
Occupation																		
State‐owned firms' employees	17.21	17.48	10.96	54.35	77.74	46.18	25.28	24.15	13.58	36.98	69.06	30.75	12.82	13.85	9.54	63.79	82.46	54.56
Private firms' employees	28.49	19.32	13.82	38.36	71.23	29.06	28.30	19.94	14.47	37.30	67.20	28.94	28.64	18.84	13.32	39.20	74.37	29.15
Self‐employed	21.78	20.44	14.07	43.72	74.37	36.01	26.34	25.51	14.81	33.33	70.37	26.75	18.64	16.95	13.56	50.85	77.12	42.37
Professional technical personnel	6.66	11.20	5.90	76.25	86.76	69.82	14.94	19.92	6.22	58.92	78.01	51.87	4.81	9.25	5.83	80.11	88.71	73.82
Farmers or herders	27.53	21.60	14.20	36.67	71.98	30.74	34.52	24.52	13.55	27.42	67.10	20.97	23.20	19.80	14.60	42.40	75.00	36.80
Unemployed	33.72	18.31	15.70	32.27	75.29	25.29	42.11	15.79	12.78	29.32	75.19	23.31	28.44	19.91	17.54	34.12	75.36	26.54
Others	18.01	22.60	16.33	43.06	76.96	37.36	22.30	27.14	16.36	34.20	70.63	28.25	16.16	20.64	16.32	46.88	79.68	41.28
Annual income																		
< 10,000 CNY	23.31	19.80	11.91	44.98	73.03	37.80	28.60	25.17	12.13	34.10	67.28	27.23	20.90	17.35	11.81	49.95	75.65	42.63
10,000–50,000 CNY	18.95	19.83	12.39	48.82	79.29	43.15	29.84	24.91	11.59	33.66	73.37	28.73	13.32	17.21	12.81	56.66	82.35	50.61
> 50,000 CNY	16.74	15.04	11.59	56.63	78.56	47.99	22.56	20.03	15.72	41.70	68.82	33.33	13.91	12.61	9.58	63.91	83.31	55.13
Stroke risk factors																		
None	19.52	17.35	13.67	49.45	76.24	42.16	25.21	22.44	9.83	42.52	74.38	36.29	15.31	17.39	8.94	58.36	82.18	51.07
1 or more	18.34	18.94	9.21	53.50	79.79	46.54	27.60	23.42	15.21	33.76	68.06	26.84	15.29	14.18	12.86	57.67	80.53	50.18

^a^ The Stroke Belt regions Heilongjiang, Tibet, Jilin, Liaoning, Xinjiang, Hebei, Inner Mongolia, Beijing, and Ningxia.

### 3.3 EMS Response

**FIGURE 2:**
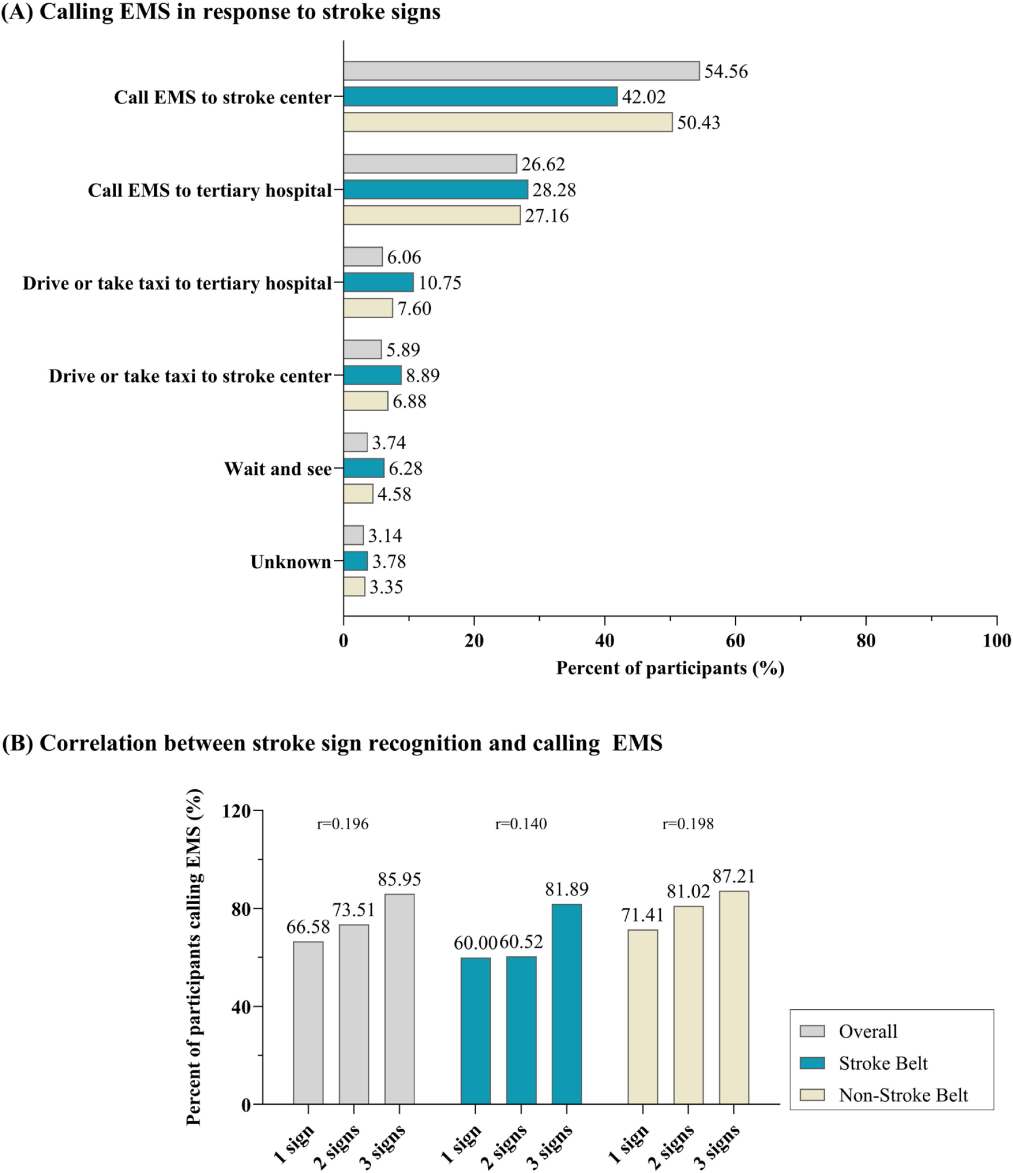
Proportion (%) of individuals who know to call EMS in the event of stroke.

Among community residents who participated in the survey, 77.59% (*n* = 4796) identified calling EMS to seek care (Table 2). The proportion of participants who identified calling EMS was lower in the Stroke Belt regions than in the non‐Stroke Belt regions (70.30% vs. 81.18%; *p* < 0.001). As shown in Figure 2, 50.43% of individuals identified calling EMS to seek care in the stroke center, followed by 27.16% of individuals who would call EMS to seek care in the tertiary hospital, 7.6% of individuals who would drive or take a taxi to a tertiary hospital, 6.88% of individuals who would drive or take a taxi to the stroke center, and 4.58% of individuals who would wait and see. The proportion of participants who called EMS to a nearby stroke center was lower in Stroke Belt regions than in non‐Stroke Belt regions (42.02% vs. 50.43%; *p* < 0.001).

As shown in Figure 2, only 24.12% of participants considered themselves very familiar with the stroke map, while 39.04% had never heard of it. The proportion of participants who considered themselves very familiar with the stroke map was lower in the Stroke Belt regions than in the non‐Stroke Belt regions (16.30% vs. 27.97%; *p* < 0.001). The percentage of participants who had never heard of the stroke map was also higher in Stroke Belt regions than in the non‐Stroke Belt regions (45.66% vs. 35.79%; *p* < 0.001).

We also examined the response to a perceived stroke in relation to awareness of stroke signs (Figure 2). The percentage of individuals who would call EMS was highest among those who recognized all 3 stroke signs (85.95%), followed by those who recognized 2 signs (73.51%) and those who recognized only 1 sign (66.58%).

### 3.4 Knowledge of Stroke Therapy

**FIGURE 3:**
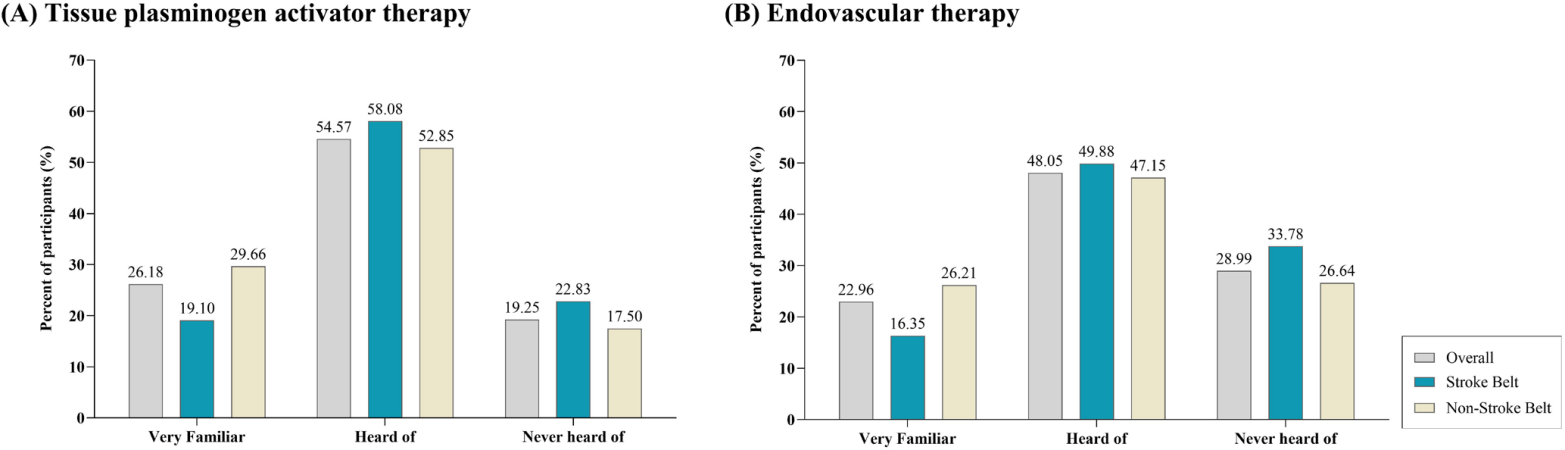
Individual's awareness of stroke treatment.

Among the community residents, only 26.18% of participants considered themselves very familiar with tPA therapy, and 19.25% of them had never heard of this therapy for stroke (Figure 3). The proportion of participants familiar with EVT therapy was similar to that for tPA: 22.96% considered themselves familiar with EVT therapy, while 28.99% had never heard of it for stroke. The proportion of participants who considered themselves very familiar with tPA and EVT therapy was lower in the Stroke Belt regions than in the non‐Stroke Belt regions.

### 3.5 Preferred Source of Information

**FIGURE 4:**
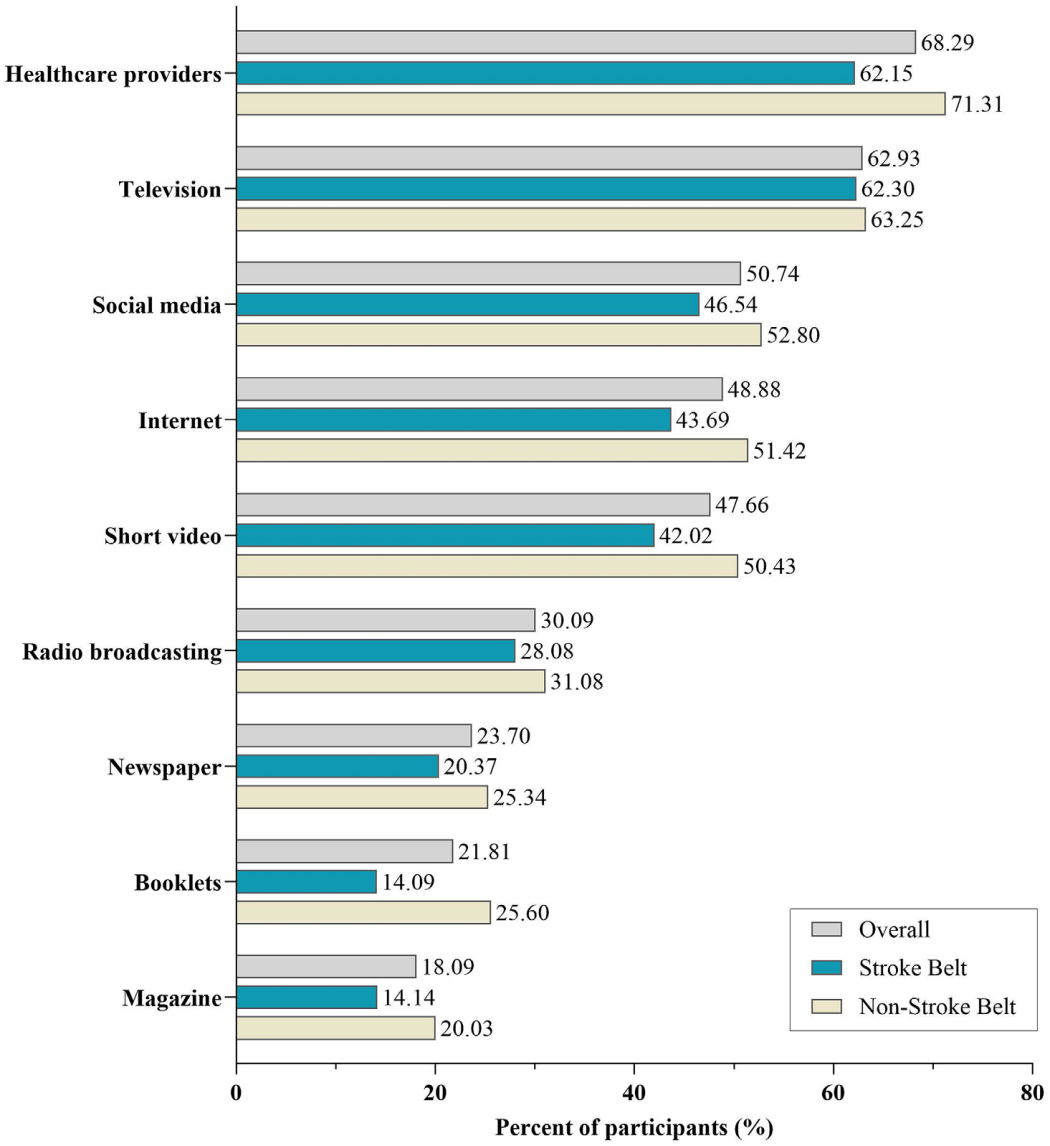
Individual's perceived source of information for stroke knowledge.

Regarding the sources for acquiring stroke‐related knowledge, the most preferred sources were healthcare providers (Figure 4; *n* = 4221; 68.29%), followed by television (*n* = 3890; 62.93%), social media (*n* = 3136; 50.74%), Internet (*n* = 3021; 48.88%), short video (*n* = 2946; 47.66%), radio broadcasting (*n* = 1860; 30.09%), newspaper (*n* = 1465; 23.7%), booklets or posters (*n* = 1348; 21.81%), and magazine (*n* = 1118; 18.09%). Participants residing in the Stroke‐Belt regions were more interested in acquiring stroke‐related knowledge than those living in the non‐Stroke Belt regions.

### 3.6 Sociodemographic and Geographic Factors Influencing Awareness and Response to Stroke

Multivariable logistic regression analysis revealed that participants residing in the Stroke Belt region had lower likelihoods of recognizing all three stroke signs (Table 3; adjusted odds ratio [aOR]: 0.50; 95% confidence interval [CI]: 0.44–0.56; *p* < 0.001), calling EMS (aOR: 0.62; 95% CI: 0.54–0.70; *p* < 0.001), and knowing all three signs and calling EMS (aOR: 0.51; 95% CI: 0.45–0.58; *p* < 0.001). In addition, significant factors of being aware of all three signs and calling EMS were being female, having higher education, and being professional technical personnel.

**TABLE 3:** Adjusted odds ratio of not being aware of stroke signs and need to call EMS.

Characteristic	Know all three symptoms		Know to call EMS		Know all three signs and to call EMS	
	OR (95% CI)	*p*	OR (95% CI)	*p*	OR (95% CI)	*p*
Stroke belt^a^						
Yes	**0.50 (0.44–0.56)**	**< 0.001**	**0.62 (0.54–0.70)**	**< 0.001**	**0.51 (0.45–0.58)**	**< 0.001**
No	Ref	—	Ref	—	Ref	—
Age, year						
18–44	Ref	—	Ref	—	Ref	—
45–59	0.99 (0.85–1.14)	0.833	0.89 (0.75–1.06)	0.199	0.96 (0.83–1.11)	0.558
60–74	0.69 (0.58–0.83)	**< 0.001**	0.68 (0.55–0.83)	**< 0.001**	0.65 (0.55–0.78)	**< 0.001**
75+	0.73 (0.54–1.00)	0.046	0.64 (0.47–0.89)	**0.007**	0.76 (0.55–1.04)	0.087
Sex						
Men	Ref	—	Ref	—	Ref	—
Women	1.36 (1.22–1.53)	**< 0.001**	1.29 (1.13–1.46)	**< 0.001**	1.30 (1.16–1.46)	**< 0.001**
Education						
Elementary school	Ref	—	Ref	—	Ref	—
Middle school	1.25 (0.92–1.69)	0.156	1.80 (1.35–2.41)	**< 0.001**	1.39 (1.00–1.94)	0.051
High school	1.93 (1.40–2.67)	**< 0.001**	2.23 (1.63–3.06)	**< 0.001**	2.07 (1.46–2.93)	**< 0.001**
College or above	3.37 (2.39–4.76)	**< 0.001**	2.64 (1.87–3.73)	**< 0.001**	3.33 (2.30–4.83)	**< 0.001**
Marriage						
Married	Ref	—	Ref	—	Ref	—
Divorced/widowed	0.84 (0.67–1.05)	0.126	0.49 (0.40–0.62)	**< 0.001**	0.71 (0.56–0.91)	**0.007**
Never married	0.94 (0.77–1.14)	0.515	0.66 (0.53–0.82)	**< 0.001**	0.84 (0.70–1.02)	0.079
Occupation						
Employees of state‐owned firms/institutions	Ref	—	Ref	—	Ref	—
Employees of private firms/institutions	0.64 (0.53–0.78)	**< 0.001**	0.78 (0.63–0.96)	**0.019**	0.57 (0.47–0.70)	**< 0.001**
Self‐employed	0.86 (0.70–1.06)	0.166	0.92 (0.73–1.16)	0.476	0.84 (0.68–1.04)	0.107
Professional technical personnel	1.99 (1.68–2.36)	**< 0.001**	1.48 (1.20–1.82)	**< 0.001**	2.03 (1.72–2.39)	**< 0.001**
Farmers or herders	0.96 (0.77–1.19)	0.699	1.23 (0.97–1.56)	0.088	0.98 (0.79–1.22)	0.863
Unemployed	0.66 (0.50–0.88)	**0.004**	1.36 (1.00–1.86)	**0.048**	0.64 (0.48–0.85)	**0.002**
Others	0.67 (0.56–0.81)	**< 0.001**	1.02 (0.82–1.27)	0.841	0.73 (0.61–0.88)	**0.001**
Annual income						
< 10,000 CNY	Ref	—	Ref	—	Ref	—
10,000–50,000 CNY	1.05 (0.90–1.23)	0.505	1.30 (1.10–1.55)	**0.003**	1.12 (0.96–1.31)	0.140
> 50,000 CNY	1.09 (0.93–1.29)	0.288	1.12 (0.93–1.34)	0.225	1.04 (0.88–1.22)	0.660
Having any stroke risk factor	1.14 (1.01–1.29)	**0.028**	1.01 (0.88–1.15)	0.927	1.10 (0.98–1.24)	0.116

*Note:* Bold values indicate statistical significance at *p* < 0.05.

^a^ The Stroke Belt regions Heilongjiang, Tibet, Jilin, Liaoning, Xinjiang, Hebei, Inner Mongolia, Beijing, and Ningxia.

## 4 Discussion

According to this national survey on stroke action awareness among community residents in China, fewer than half of the participants could identify all three signs of a stroke and knew to call EMS when faced with a perceived stroke. The study also revealed significant differences in stroke awareness among residents living in the Stroke Belt and non‐Stroke Belt regions. Although awareness has improved in China, it still lags behind high‐income countries, where nearly 70% of the population is aware of the signs of a stroke in some high‐income countries, for example, the United States [25], Spain [23], and Norway [24]. Our findings underscore the urgent need for the design and implementation of targeted public health interventions and educational campaigns specifically aimed at increasing stroke action awareness among high‐risk populations in regions with a high incidence of stroke.

Evidence from this study indicates a significant gap in stroke action awareness [8], the biggest barrier impeding timely response and effective management during acute stroke onset, particularly in Stroke Belt regions. As innovative therapies for acute stroke have become available, reducing prehospital delay is essential to reduce stroke disability and mortality [3, 26]. This study also highlights the geographic variations in stroke action awareness, potentially explaining the elevated stroke mortality reported in the Stroke Belt regions [17, 18]. Previous studies have indicated the existence of a “stroke belt” in northern and western China [17]. Our data further indicate that Stroke Belt regions exhibit the lowest levels of stroke knowledge and awareness compared to other regions. Recent data suggested that low health literacy is generally associated with poor health status and higher mortality [27]. Therefore, the knowledge gap reported in this study underscores the urgency of well‐designed educational campaigns in Stroke Belt regions to improve stroke awareness [28]. In addition, community residents identified as having a higher risk of stroke demonstrated lower action awareness than individuals without any stroke risk factors. Hence, it is imperative to implement targeted educational campaigns for high‐risk populations in Stroke Belt regions to facilitate the identification of early stroke signs and prompt responses.

This national survey also identified sociodemographic factors as significant determinants of stroke awareness [25, 29]. First, older adults, particularly those residing in Stroke Belt regions, exhibited the lowest awareness of stroke symptoms, with men and women displaying varying levels of awareness and responsiveness to stroke events, consistent with existing literature [30, 31]. Second, cultural and societal norms shaping gender roles may affect the dissemination and reception of health information within communities, potentially leading to differing levels of engagement in health education activities between genders. Third, educational attainment emerged as a crucial factor; individuals with higher levels of education demonstrated greater awareness of stroke symptoms. Finally, disparities in stroke awareness based on income level were evident, as individuals in high‐income groups generally possessed a better grasp of stroke‐related information compared to their low‐income counterparts [31]. This discrepancy is likely linked to increased access to health services, health information, and related support systems. Stroke Belt regions are often located in underdeveloped areas of China, potentially contributing to the differences in stroke awareness. Therefore, future efforts should specifically target sociodemographic groups, particularly in Stroke Belt regions, to reduce stroke mortality and morbidity.

This study has several limitations. First, the questionnaire was developed based on the three stroke signs outlined in the Stroke 1‐2‐0 tool. Although we employed an expert panel and a group of community residents to pilot the survey, the instrument has not been validated formally. Furthermore, because the survey used nonprobability convenience and snowball sampling, selection bias may be present. Despite our efforts to recruit community residents across China, the respondents represented only a small proportion of the Chinese population. Consequently, our findings may not be generalizable to residents who did not participate in the survey. For instance, over 70% of participants were under 60 years old, and more than half were women, which differs from the demographic profile of stroke patients; this discrepancy suggests that the knowledge levels reported in this study may not accurately represent those among stroke patients. Finally, selection bias may be present, as residents who were confident in their stroke knowledge or actively sought health information were more likely to participate. Therefore, we cannot exclude the possibility that community residents with greater awareness of strokes were included.

## 5 Conclusion

This study reveals that stroke action awareness among community residents is still notably low in China. Furthermore, significant sociodemographic and geographic disparities exist in stroke action awareness. Our findings underscore the urgent need for targeted interventions to improve awareness of stroke signs and facilitate early access to emergency stroke care in China, particularly for individuals residing in the Stroke Belt.

## Funding

This work was supported by The National Nature Science Foundation of China (82173646, 82373703); Public Health Discipline Construction project of Shanghai Minhang District Health Commission (MGWXK2023‐04); University of Macau (UMDF‐TISF‐I/2026/019/ICMS, SRG2025‐00027‐ICMS); Science and Technology Development Fund, Macau SAR (File no. 0002/2025/NRP and 0008/2025/EQP).

## Conflicts of Interest

The authors declare no conflicts of interest.

## Supporting information


**Figure S1:** Schematic location of the Chinese Stroke Belt regions used in this study*.

## Data Availability

The data that support the findings of this study are available on request from the corresponding author. The data are not publicly available due to privacy or ethical restrictions.

## References

[1] “Global, Regional, and National Burden of Household Air Pollution, 1990‐2021: A Systematic Analysis for the Global Burden of Disease Study 2021,” Lancet 405, no. 10485 (2025): 1167–1181. 40118081 10.1016/S0140-6736(24)02840-X PMC11971481

[2] Z. Luo , S. Shan , J. Cao , et al., “Temporal Trends in Cross‐Country Inequalities of Stroke and Subtypes Burden From 1990 to 2021: A Secondary Analysis of the Global Burden of Disease Study 2021,” EClinicalMedicine 76 (2024): 102829. 39309727 10.1016/j.eclinm.2024.102829 PMC11415963

[3] K. Fassbender , S. Walter , I. Q. Grunwald , et al., “Prehospital Stroke Management in the Thrombectomy Era,” Lancet Neurology 19, no. 7 (2020): 601–610. 32562685 10.1016/S1474-4422(20)30102-2

[4] K. Fassbender , C. Balucani , S. Walter , S. R. Levine , A. Haass , and J. Grotta , “Streamlining of Prehospital Stroke Management: The Golden Hour,” Lancet Neurology 12, no. 6 (2013): 585–596. 23684084 10.1016/S1474-4422(13)70100-5

[5] J. Yuan , Z. K. Lu , M. Li , et al., “Late‐Time Window Endovascular Treatment Is Associated With Neurological Improvement: Evidence From the National Stroke Registry Data in China,” CNS Neuroscience & Therapeutics 30, no. 2 (2024): e14572. 38421137 10.1111/cns.14572 PMC10850790

[6] M. Bouckaert , R. Lemmens , and V. Thijs , “Reducing Prehospital Delay in Acute Stroke,” Nature Reviews. Neurology 5, no. 9 (2009): 477–483. 19668246 10.1038/nrneurol.2009.116

[7] J. Yuan , Z. K. Lu , X. Xiong , et al., “Age and Geographic Disparities in Acute Ischaemic Stroke Prehospital Delays in China: A Cross‐Sectional Study Using National Stroke Registry Data,” Lancet Regional Health Western Pacific 33 (2023): 100693. 37181525 10.1016/j.lanwpc.2023.100693 PMC10166992

[8] R. Liu , J. Zhao , and A. G. Rudd , “From Stroke Awareness to Stroke Action Awareness,” Lancet Neurology 24, no. 2 (2025): 96. 10.1016/S1474-4422(24)00527-1 39862891

[9] J. Yang , M. Zheng , S. Cheng , et al., “Knowledge of Stroke Symptoms and Treatment Among Community Residents in Western Urban China,” Journal of Stroke and Cerebrovascular Diseases 23, no. 5 (2014): 1216–1224. 24274934 10.1016/j.jstrokecerebrovasdis.2013.10.019

[10] J. Zhao and R. Liu , “Stroke 1‐2‐0: A Rapid Response Programme for Stroke in China,” Lancet Neurology 16, no. 1 (2017): 27–28. 10.1016/S1474-4422(16)30283-6 PMC5578829 28029517

[11] X. Li , Y. Liu , A. Vrudhula , R. Liu , and J. Zhao , “Middle School Students Effectively Improve Stroke Knowledge and Pass Them to Family Members in China Using Stroke 1‐2‐0,” Frontiers in Neurology 11 (2020): 203. 32322233 10.3389/fneur.2020.00203 PMC7156590

[12] Y. Liu , D. Wang , M. Chu , et al., “Value of the Stroke 1‐2‐0 Prehospital Stroke Education System: The Experience of a General Practitioner Team,” BMC Neurology 23, no. 1 (2023): 431. 38062426 10.1186/s12883-023-03476-0 PMC10770900

[13] J. Yuan , M. Li , Y. Liu , et al., “Analysis of Time to the Hospital and Ambulance Use Following a Stroke Community Education Intervention in China,” JAMA Network Open 5, no. 5 (2022): e2212674. 35579896 10.1001/jamanetworkopen.2022.12674 PMC9115614

[14] J. Yuan , G. L. Shan , S. D. Li , C. P. Gao , L. Y. Cui , and B. Peng , “Impact of Regional Differences in Stroke Symptom Awareness and Low‐Income Status on Seeking Emergency Medical Service in China,” Chinese Medical Journal 134, no. 15 (2021): 1812–1818. 34397585 10.1097/CM9.0000000000001604 PMC8367037

[15] S. Li , L. Y. Cui , C. Anderson , et al., “Public Awareness of Stroke and the Appropriate Responses in China: A Cross‐Sectional Community‐Based Study (FAST‐RIGHT),” Stroke 50, no. 2 (2019): 455–462. 32125134 10.1161/STROKEAHA.118.023317

[16] X. Wu , T. Somdee , and S. Yangyuen , “Stroke‐Related Knowledge and Warning Signs and Its Association With Seeking Emergency Medical Service Among Urban and Rural Residents in Huanggang, China,” Journal of Education Health Promotion 14 (2025): 222. 40575505 10.4103/jehp.jehp_1637_24 PMC12200242

[17] G. Xu , M. Ma , X. Liu , and G. J. Hankey , “Is There a Stroke Belt in China and Why?,” Stroke 44, no. 7 (2013): 1775–1783. 23674531 10.1161/STROKEAHA.113.001238

[18] G. Howard and V. J. Howard , “Twenty Years of Progress Toward Understanding the Stroke Belt,” Stroke 51, no. 3 (2020): 742–750. 32078485 10.1161/STROKEAHA.119.024155

[19] G. J. Hankey , “Stroke,” Lancet 389, no. 10069 (2017): 641–654. 27637676 10.1016/S0140-6736(16)30962-X

[20] Y. Zhao , L. Yang , R. Tan , and J. Yuan , “Evaluation of the Knowledge of and Attitudes Towards Pharmacovigilance Among Healthcare Students in China: A Cross‐Sectional Study,” BMC Medical Education 24, no. 1 (2024): 570. 38789989 10.1186/s12909-024-05561-5 PMC11127336

[21] L. He , C. Lv , X. Song , et al., “Knowledge and Practices Related to Snakebite Prevention, China: A Cross‐Sectional Study,” Bulletin of the World Health Organization 102, no. 4 (2024): 234–243. 38562205 10.2471/BLT.23.290169 PMC10976863

[22] M. Naderifar , H. Goli , and F. Ghaljaie , “Snowball Sampling: A Purposeful Method of Sampling in Qualitative Research,” Strides in Development of Medical Education 14, no. 3 (2017): 1–6.

[23] K. Lundelin , A. Graciani , J. García‐Puig , et al., “Knowledge of Stroke Warning Symptoms and Intended Action in Response to Stroke in Spain: A Nationwide Population‐Based Study,” Cerebrovascular Diseases 34, no. 2 (2012): 161–168. 22907330 10.1159/000341408

[24] K. W. Faiz , A. Sundseth , B. Thommessen , and O. M. Rønning , “Patient Knowledge on Stroke Risk Factors, Symptoms and Treatment Options,” Vascular Health and Risk Management 14 (2018): 37–40. 29445287 10.2147/VHRM.S152173 PMC5808699

[25] S. L. Jackson , B. Legvold , A. Vahratian , et al., “Sociodemographic and Geographic Variation in Awareness of Stroke Signs and Symptoms Among Adults—United States, 2017,” MMWR. Morbidity and Mortality Weekly Report 69, no. 44 (2020): 1617–1621. 33151923 10.15585/mmwr.mm6944a1 PMC7643899

[26] S. Man , N. Solomon , B. Mac Grory , et al., “Shorter Door‐To‐Needle Times Are Associated With Better Outcomes After Intravenous Thrombolytic Therapy and Endovascular Thrombectomy for Acute Ischemic Stroke,” Circulation 148, no. 1 (2023): 20–34. 37199147 10.1161/CIRCULATIONAHA.123.064053 PMC10356148

[27] N. D. Berkman , S. L. Sheridan , K. E. Donahue , D. J. Halpern , and K. Crotty , “Low Health Literacy and Health Outcomes: An Updated Systematic Review,” Annals of Internal Medicine 155, no. 2 (2011): 97–107. 21768583 10.7326/0003-4819-155-2-201107190-00005

[28] M. Rasura , M. Baldereschi , A. Di Carlo , et al., “Effectiveness of Public Stroke Educational Interventions: A Review,” European Journal of Neurology 21, no. 1 (2014): 11–20. 24102755 10.1111/ene.12266

[29] R. Mszar , S. Mahajan , J. Valero‐Elizondo , et al., “Association Between Sociodemographic Determinants and Disparities in Stroke Symptom Awareness Among US Young Adults,” Stroke 51, no. 12 (2020): 3552–3561. 33100188 10.1161/STROKEAHA.120.031137

[30] B. Rioux , V. Brissette , F. F. Marin , P. Lindsay , M. R. Keezer , and A. Y. Poppe , “The Impact of Stroke Public Awareness Campaigns Differs Between Sociodemographic Groups,” Canadian Journal of Neurological Sciences 49, no. 2 (2022): 231–238. 10.1017/cjn.2021.76 33875043

[31] T. E. Madsen , K. A. Baird , B. Silver , and A. Gjelsvik , “Analysis of Gender Differences in Knowledge of Stroke Warning Signs,” Journal of Stroke and Cerebrovascular Diseases 24, no. 7 (2015): 1540–1547. 25899157 10.1016/j.jstrokecerebrovasdis.2015.03.017

