# Supplementary material for: Geographic Disparities in Stroke Action Awareness in China: A National Survey
Source: CNS Neurosci Ther. 2026 Aug 4;32(8):e71056. doi: 10.1002/cns.71056 (PMC13435099; doi:10.1002/cns.71056)
Supplement: Supplementary file 1 [file CNS-32-e71056-s001.docx]

**Supplementary Figure I. Schematic location of the Chinese Stroke Belt regions used in this study***

The red-colored shaded regions indicate stroke-belt regions, including Heilongjiang, Jilin, Liaoning province, Hebei, Beijing, Inner Mongolia, Ningxia, Xinjiang, and Tibet. The map is provided only to help international readers visualize the geographic location of the Stroke Belt.
